# Quantum Confined One-Dimensional Lepidocrocite TiO_2_–Assisted In Situ Synthesis of ZIF‑8 MOF: Hierarchical Porous Nanocomposites Exhibit Improved Li-ion Transport

**DOI:** 10.1021/acsmaterialsau.6c00110

**Published:** 2026-10-16

**Authors:** Takayuki Kono, Avishek Karmakar, Keita Tsubakimoto, Kaustubh Sudhakar, Neal A. Cardoza, Vibha Kalra, Tarek Ali El-Melegy, Kota Ota, Mary Qin Hassig, Takashi Kanazawa, Hiroyuki Morita, Shun Sakaida, Takeshi Torita, Michel W. Barsoum

**Affiliations:** † Murata Manufacturing Co., Ltd, Nagaokakyo-shi, Kyoto 617-8555, Japan; ‡ Department of Materials Science and Engineering Drexel University, Philadelphia, Pennsylvania 19104, United States; § Department of Chemical and Biological Engineering, Drexel University, 3141 Chestnut Street, Philadelphia, Pennsylvania 19104, United States; ∥ Smith School of Chemical and Biomolecular Engineering, Cornell University, Ithaca, New York 14853, United States; ⊥ Department of Mechanical Engineering, The British University in Egypt, Cairo 1837, Egypt

**Keywords:** one-dimensional titanates, ZIF-8 metal organic framework, lepidocrocite, composite, hierarchical pores, lithium−sulfur battery

## Abstract

One-dimensional (1D) nanostructured materials offer distinctive physical and chemical properties suitable for advanced energy applications. Among these, 1D lepidocrocite-type TiO_2_ (1DL), recently discovered, has shown potential in batteries, water splitting, wastewater treatment, and polymer composites, among others. In this study, we report on the combination of 1DL with an in situ formed metal–organic framework (MOF), specifically ZIF-8, to produce a hierarchically porous ZIF-8/1DL composite. X-ray diffraction (XRD) confirms the successful in situ formation of ZIF-8 in the presence of 1DL. In the composite, the average ZIF-8 size is ∼30 nm, that is smaller than that observed in conventional bulk syntheses that typically yield micron-scale crystals. Zeta-potential measurements indicate electrostatic interactions between positively charged ZIF-8 particles and negatively charged 1DL, which stabilize the composite. Transmission electron microscopy (TEM) and energy-dispersive X-ray spectroscopy (EDS) mapping show a relatively uniform distribution of ZIF-8 on 1DL surfaces. Nitrogen adsorption–desorption analyses reveal a substantial increase in specific surface area from 20 m^2^ g^–1^ for 1DL to 501 m^2^ g^–1^ for ZIF-8/1DL, together with the coexistence of micropores and newly generated mesopores. Electrochemical evaluation in lithium–sulfur (Li–S) batteries indicates enhanced Li-ion transport in the composite compared with 1DL alone. These findings highlight the utility of 1DL as a structural framework for MOF-based nanocomposites and provide a pathway to hierarchical designs for energy-storage applications.

## Graphical abstract

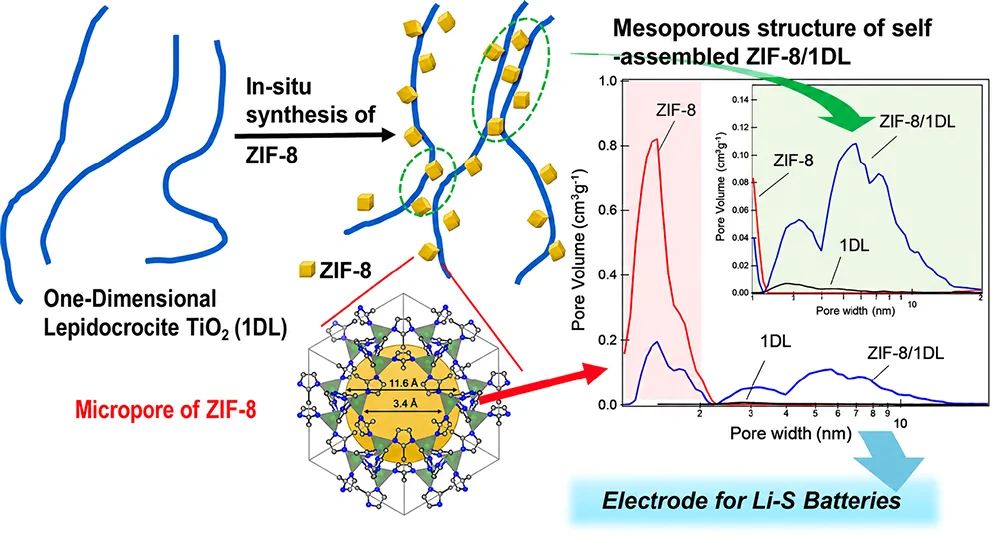

## Introduction

Among the many materials engineered into one-dimensional (1D) architectures, titanium dioxide (TiO_2_) stands out due to its chemical stability, low toxicity, and strong photocatalytic capabilities.^1–4^ Its versatility is evident in applications ranging from electron-transport layers in photovoltaics to photocatalytic degradation of organic pollutants in environmental remediation.^2,3^

We recently reported on quantum-confined, one-dimensional lepidocrocite-type TiO_2_ structures—hereafter referred to as 1DLs—that enable mass production of 1D titanate under near-ambient conditions.^5,6^ In contrast to the high-temperature, high-pressure hydrothermal routes pioneered by Kasuga et al.,^7^ our approach proceeds at atmospheric pressure and modest temperatures. The uniqueness of 1DLs lies in their cross-sectional dimensions (≈5 × 7 Å^2^), among the smallest reported for titanate-based 1D units. To put this in perspective, 1 g of 1DL placed end to end would span roughly 600 million km. The fundamental building block is an inorganic TiO_2_ polymer chain (in cross-section, tens of nanometers long; Figure 1A).^6,8^ Upon drying, these nanofilaments (NFs) self-assemble into diverse morphologies—including porous mesoscale particles (PMPs), two-dimensional (2D) flakes, and 1D nanoribbons—depending on washing and drying solvents used during processing.^8–13^

**Figure 1:**
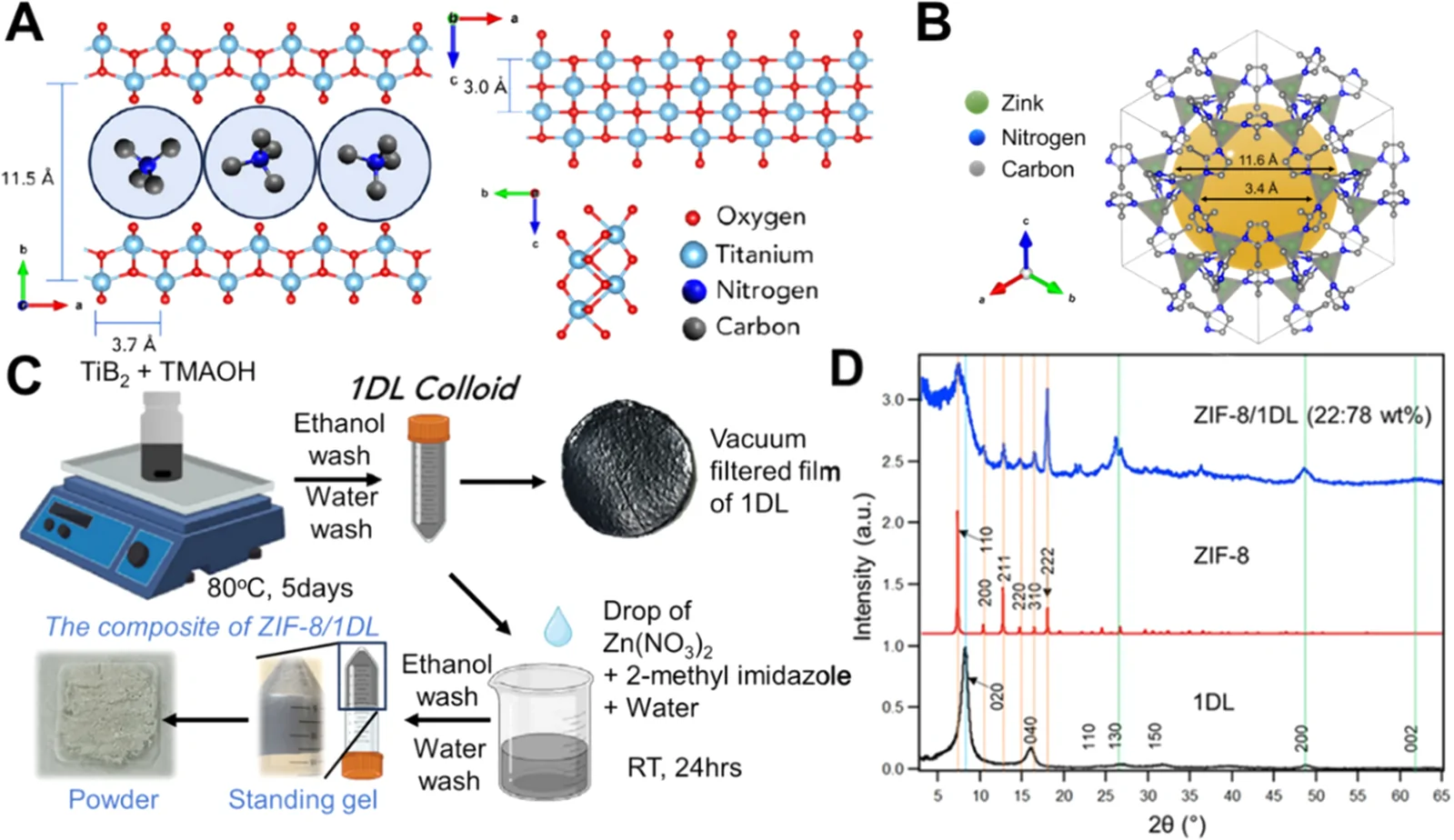
(A) DFT-predicted 1DL structure. (B) Structure of ZIF-8 with pore-size information. (C) Diagram of the ZIF-8/1DL synthesis. (D) XRD patterns for crushed filtered films of 1DL, ZIF-8, and ZIF-8/1DL.

The 1D morphology imparts properties advantageous in several applications, such as water splitting and batteries.^14,15^ A notable feature is the unusually large band gap energy (*E*_g_ ≈ 4.0 eV), attributed to quantum confinement in the 1D structure.^16,17^ Walter et al. further reported *E*_g_ values up to ≈4.5 eV in 1DL filtered films, FFs, by decreasing the concentration of the colloidal suspensions used to prepare the films, implying enhanced confinement upon dilution as the system transitions from 2D to 1D behavior.^18^ Another important attribute is the remarkable adsorption capacity and selectivity toward organic dyes,^19,20^ which exceeds that of activated carbon or high-capacity clays, consistent with large effective specific surface areas, SSAs, arising from low dimensionality.^21,22^ It is worth noting that Brunauer–Emmett–Teller (BET) surface areas derived from N_2_ adsorption are, for 1DLs and related nanomaterials (e.g., MXenes), often at least an order of magnitude lower than effective areas inferred from adsorption experiments. A back-of-the-envelope estimate yields ≈1500 m^2^ g^–1^ for 1DL, consistent with our dye-adsorption results.^21^

Metal–organic frameworks (MOFs) are crystalline materials formed by self-assembly of inorganic nodes and organic linkers.^23–25^ They exhibit high SSAs tunable pore sizes, and modular design, supporting applications in gas storage, catalysis, drug delivery, and chemical sensing.^23,26–30^ Among MOFs, zeolitic imidazolate frameworks (ZIFs) are notable for their exceptional thermal and chemical stability, with ZIF-8 being a well-studied example featuring a three-dimensional microporous network.^31,32^

Lithium–sulfur (Li–S) batteries offer a theoretical energy density of ≈2600 Wh kg^–1^,^33^ yet deployment is hindered by the low electronic conductivity of sulfur, S, formation, shuttling of soluble lithium polysulfides (LiPS), and challenges associated with the use of Li-metal anodes.^34^ Microporous hosts such as ZIF-8 physically confine S and LiPS within their pore frameworks, mitigating shuttle effects to some extent.^35–37^ However, due to limited polar or chemically active sites, ZIF-8 primarily affords weak physical adsorption, which can compromise long-term cycling stability.^35,36,38^

To enhance LiPS–host interactions, polar materials such as TiO_2_ and MnO_2_ have been incorporated to introduce strong chemical binding sites, improving cycling performance and Coulombic efficiency.^39–43^ Conductive porous carbon frameworks (e.g., carbon nanotubes and graphene oxide) have likewise been explored to simultaneously provide physical confinement and partial chemical adsorption.^44–47^ Recent work shows that cathodes incorporating 1DL leverage exposed Ti^4+^ sites to form Lewis acid–base interactions with LiPS, effectively anchoring them and suppressing diffusion, thereby enabling stable capacities (≈750 mAh g^–1^ over 300 cycles) with nearly 100% Coulombic efficiency.^48^

Introducing mesoporosity can further facilitate rapid ion transport while suppressing LiPS dissolution.^49,50^ Blending ZIF-8 with mesoporous components promotes faster Li^+^ diffusion and reduces LiPS dissolution, improving overall performance.^37,49,51,52^ Thus, from the perspectives of long-term cycling stability and high-rate capability, a mesoporous ZIF-8 composite could outperform ZIF-8 alone. If the composite also contains 1DL, it can combine strong Lewis acid–base interactions with hierarchical porosity.

Guided by these considerations, we hypothesized that low-dimensional 1DL, when combined with a MOF, would yield a hierarchically porous material. Because 1DLs are negatively charged near neutral pH, they should form composites with positively charged ZIF-8 particles.^9^ To simplify processing, we chose to synthesize ZIF-8 in situ in a 1DL dispersion. Alternative strategies—synthesizing each component separately and blending, or synthesizing 1DL in the presence of ZIF-8—are less favorable due to dispersion challenges (ZIF-8 in water) and base sensitivity of ZIF-8, respectively. We further anticipated that in situ growth would promote uniform ZIF-8 distribution on the 1DLs because Zn^2+^ precursors could be stabilized on negatively charged 1DL surfaces via electrostatic attraction.^9^

Herein, we demonstrate a ZIF-8/1DL composite formed by in situ synthesis of ZIF-8 within a 1DL colloid. We select ZIF-8 for its well-defined microporosity, high SSA, and demonstrated utility in gas separation and adsorption. ZIF-8’s metal centers support interactions with S species, while its porous framework enables uniform S distribution and facilitates Li-ion transport.^53^ Combining ZIF-8 with 1DL aims to further improve electrochemical behavior through hierarchical porosity and chemical affinity.

## Results and Discussion

### Confirmation of ZIF-8 Nucleation and Growth in the Presence of 1DL

Figure 1A reproduces the density functional theory (DFT)–predicted 1DL structure. The ZIF-8 structure, with pore-size information from the literature is shown in Figure 1B.^55,56^ XRD patterns of crushed FFs for 1DL, ZIF-8, and ZIF-8/1DL (22:78 wt %) are shown in Figure 1D (2θ = 3–65°). The ZIF-8/1DL ratio, determined by ICP-AES, is listed in Table S1; the C_8_H_12_N_4_Zn:TiO_2_ molar ratio is 9:91. The 1DL pattern exhibits typical 0k0 reflections,^21,57^ with a 2θ ≈ 7.5° peak assigned to the 020 basal reflection (*d*_020_ ≈ 11.7 Å; dashed blue line). This *d*-spacing depends on intercalated cation size.^9^ Peaks near 48° and 62° (dashed green lines) correspond to the 200 and 002 reflections of the lepidocrocite structure, respectively, and are independent on interlayer cations.^9^ The ZIF-8 pattern (middle trace) shows peaks at 2θ = 7.4°, 10.4°, 12.7°, 14.9°, 16.5°, and 18.2°, indexed to the (110), (200), (211), (220), (310), and (222) planes.^55^

The composite pattern (top trace) contains reflections from both phases. The peak at 2θ = 7.4° is substantially broadened due to overlap of the 1DL 020 and ZIF-8 110 peaks and to intrinsic broadening of the ZIF-8 reflections. Scherrer analysis (Figure S1) gives a ZIF-8 crystallite size of 31 ± 9 nm. In contrast, the narrow ZIF-8 peaks in the standalone sample preclude reliable size estimation. These observations indicate that ZIF-8 growth is constrained within the 1DL gel network (Figure 2B),^58–60^ which limits particle coarsening. Notably, the positions of the 200 and 002 reflections of 1DL are unchanged in the composite.

**Figure 2:**
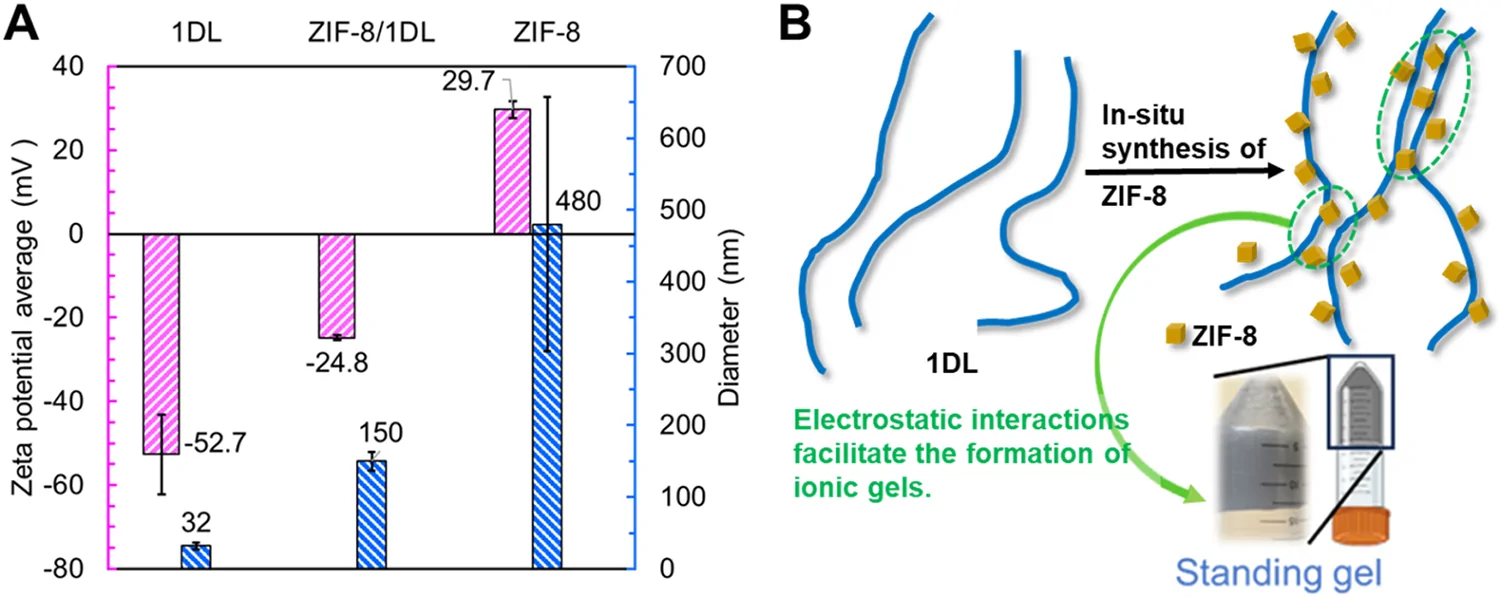
(A) Zeta potential (left axis) and average hydrodynamic diameter (right axis) of 1DL, ZIF-8/1DL, and ZIF-8 at pH ≈ 7. (B) Schematic of ionic-gel formation via electrostatic interactions between ZIF-8 and 1DLs.

### Interfacial Interactions and Composite Morphology

Figure 2A presents zeta potentials, ζ, (pink bars, left axis) and hydrodynamic diameters, h_d_, (blue bars, right axis) of crushed FFs diluted 10× in water. The ζ values of ZIF-8 and 1DL are +29.7 mV and −52.7 mV, respectively, indicating opposite charges at pH ≈ 7. The composite exhibits an intermediate ζ (−24.8 mV) and good monodispersity (Figure S2). Upon water washing, the composite forms a gel (Figure 2B), most likely an ionic gel formed by electrostatic interactions, analogous to recently reported 1DL/acid gels.^13^ While such gels may be useful in their wet state, that is beyond the scope of this work.^61,62^

The average h_d_ values are ≈32 ± 5 nm for 1DL, ≈150 ± 13 nm for ZIF-8/1DL, and ≈480 ± 177 nm for ZIF-8 alone. The 1DL size agrees with prior reports.^11^ Two concurrent effects are observed: adding ZIF-8 to 1DL increases agglomerate size by approximately a factor of 5, whereas adding 1DL to ZIF-8 reduces agglomerate size by roughly a factor of 3. Note the h_d_ values for 1DL and ZIF-8/1DL should be interpreted with caution because the Smoluchowski model assumes spherical particles. This comment notwithstanding, it is reasonable to conclude that, the composite agglomerate size lies between those of the two end members.

Figure 3 shows high resolution XPS spectra of the composite. In the Ti 2p region, doublets at binding energies, BEs, 458.5/464.3 eV and 457.7/463.0 eV correspond to Ti^4+^ and Ti^3+^ (2p_3_/_2_ and 2p_1_/_2_) cations, consistent with other 1DL samples reported previously.^5,63^ The Zn 2p_3_/_2_ peak at BE 1021.9 eV indicates Zn^2+^ from ZIF-8.^64^ In C 1s, the C–N signal at BE 286.0 eV (Figure 3C) indicates the imidazolate linker; other C 1s components are attributed to adventitious carbon, as sputtering was avoided to preserve the 1DL structure.^65,66^ The N 1s spectrum shows an imidazole peak at BE 400.5 eV and an −NH– peak at 399.1 eV.^67,68^ In O 1s, the dominant peak at BE 529.9 eV is from metal–oxygen bonds (Ti–O and Zn–O), with additional contributions from metal–hydroxide (≈531.2 eV) and adsorbed water (≈532.9 eV).^63,69^

**Figure 3:**
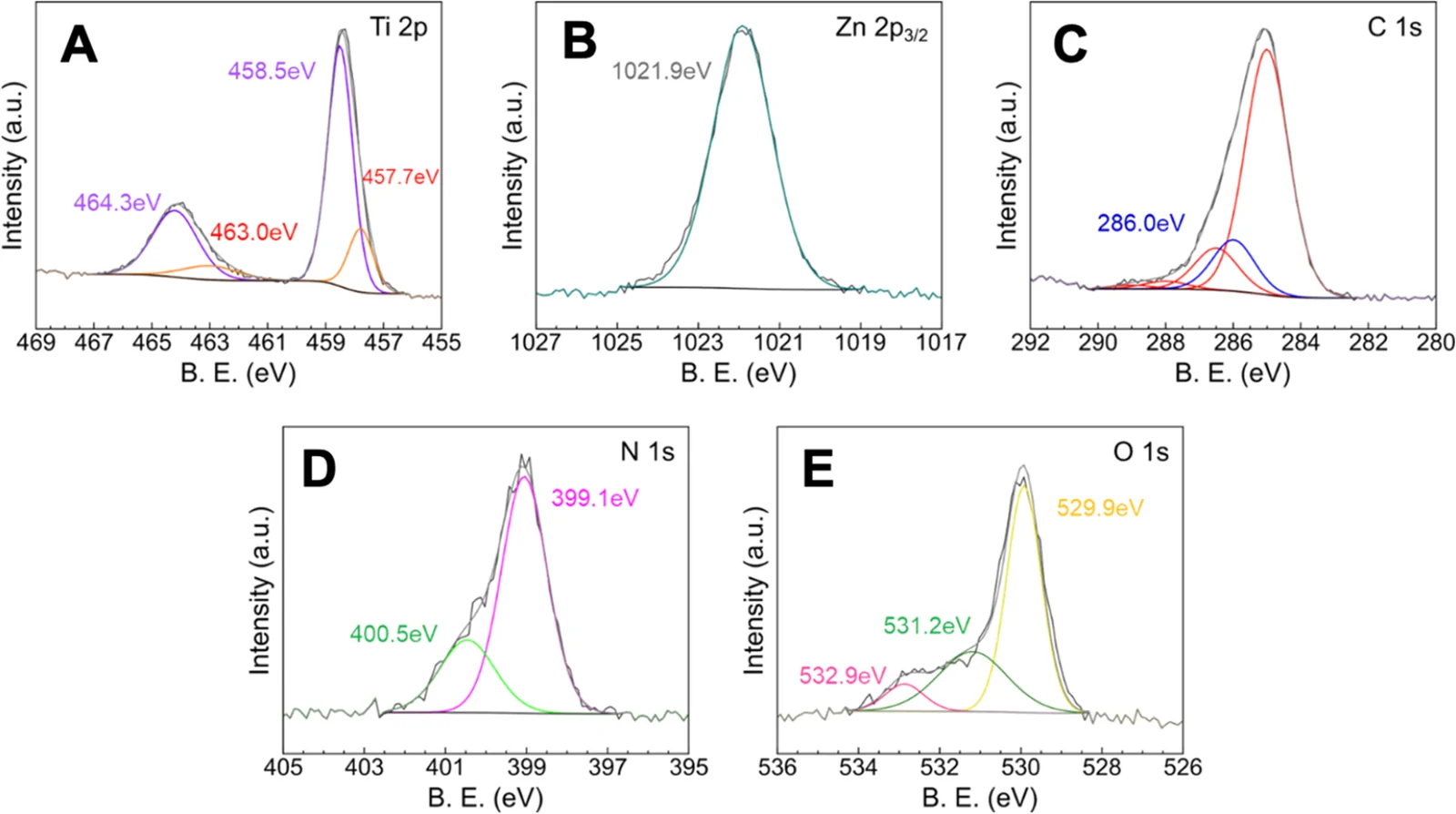
High-resolution XPS of ZIF-8/1DL composites: (A) Ti 2p, (B) Zn 2p_3_/_2_, (C) C 1s, (D) N 1s, and (E) O 1s.

Figure 4A–E present TEM micrographs, at various magnifications, of samples from diluted FF suspensions drop-cast on Cu grids. Because the starting material was a filtered colloid, the 1DLs appear as 2D sheets rather than 1D filaments.^5,11^ (For comparison, unique 1D morphology in diluted 1DL-only suspensions appears in Figure S3B.) The images show small flakes sometimes decorated with smaller particles, some hexagonal, consistent with the rhombic dodecahedral habit of ZIF-8.^54^ SAED patterns (Figure S3A) collected from the circled region in Figure 4C reveal sharp spots not associated with amorphous-like 1DL layers,^11^ corroborating the presence of crystalline ZIF-8 particles. Some particles approach ≈500 nm—larger than the Scherrer size—implying that numerous smaller particles likely exist but are more challenging to image.

**Figure 4:**
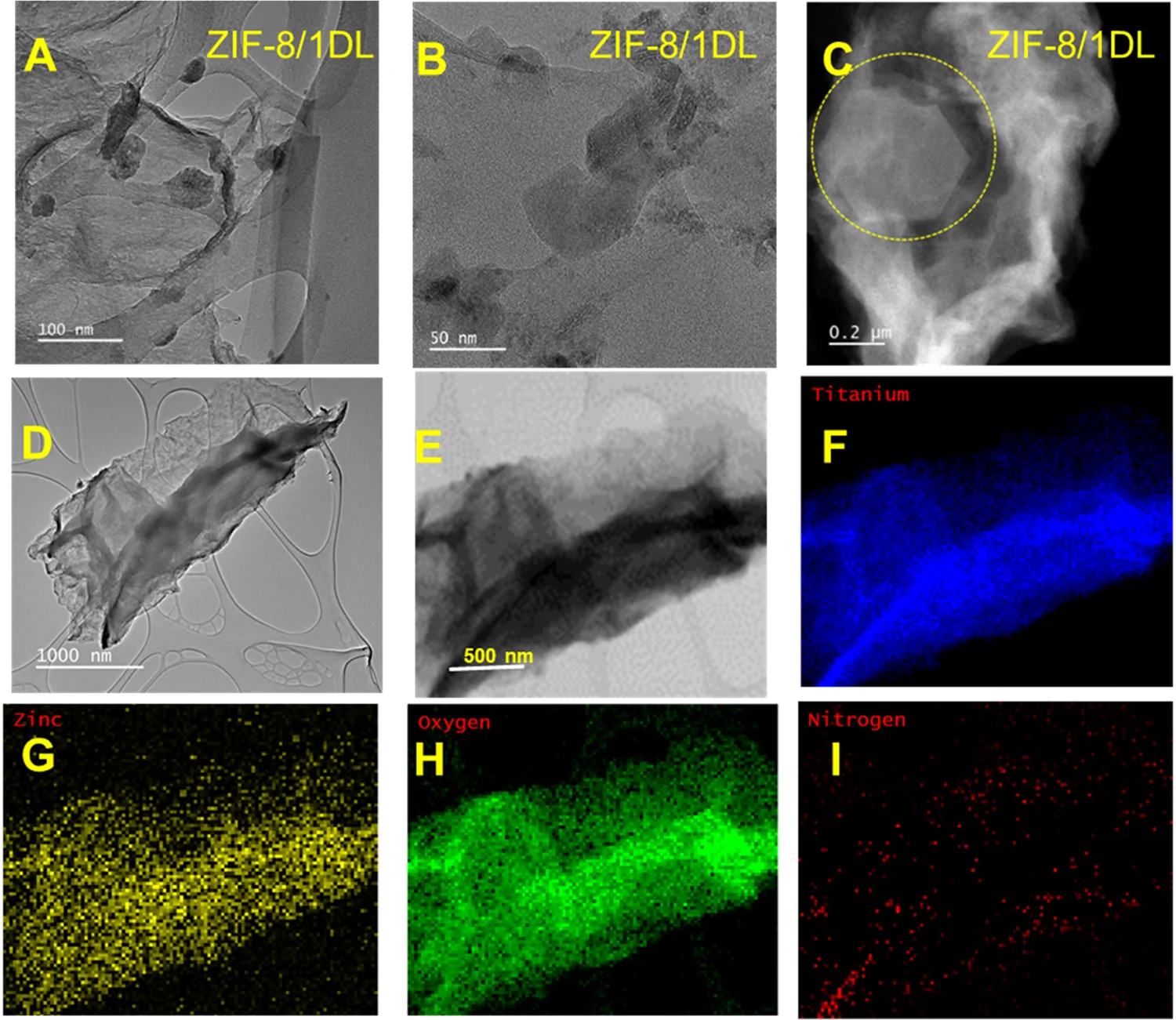
TEM of ZIF-8/1DL composites prepared from crushed FFs (diluted 1000× in water; drop-cast on Cu grids). (A) 2D flakes; (B, C) higher magnifications of panel A. SAED from circled region in panel C is in Figure S3A. (D,E) Crumpled ZIF-8/1DL sheets. (F–I) EDS elemental maps corresponding to panel E.

Figure 4D shows a typical crumpled ZIF-8/1DL sheet. Figure 4E provides a higher-magnification view. EDS elemental maps of Ti, Zn, O, and N (Figure 4F–I) confirm the presence of expected elements, with the Zn signal distributed relatively uniformly, indicating effective dispersion of ZIF-8 across 1DL surfaces.

Figure 5 presents representative SEM images. Figure 5A shows the layered surface of a 1DL FF. In the composite (Figure 5B), small particles appear on the 2D surfaces, and some particles are embedded within the layered structure (Figure 5C), consistent with self-assembly of ZIF-8/1DL into 2D sheets during filtration. Cross-sectional images (Figure 5D–F) reveal ZIF-8 particles (<100 nm in diameter) distributed throughout the 1DL-derived layered framework.

**Figure 5:**
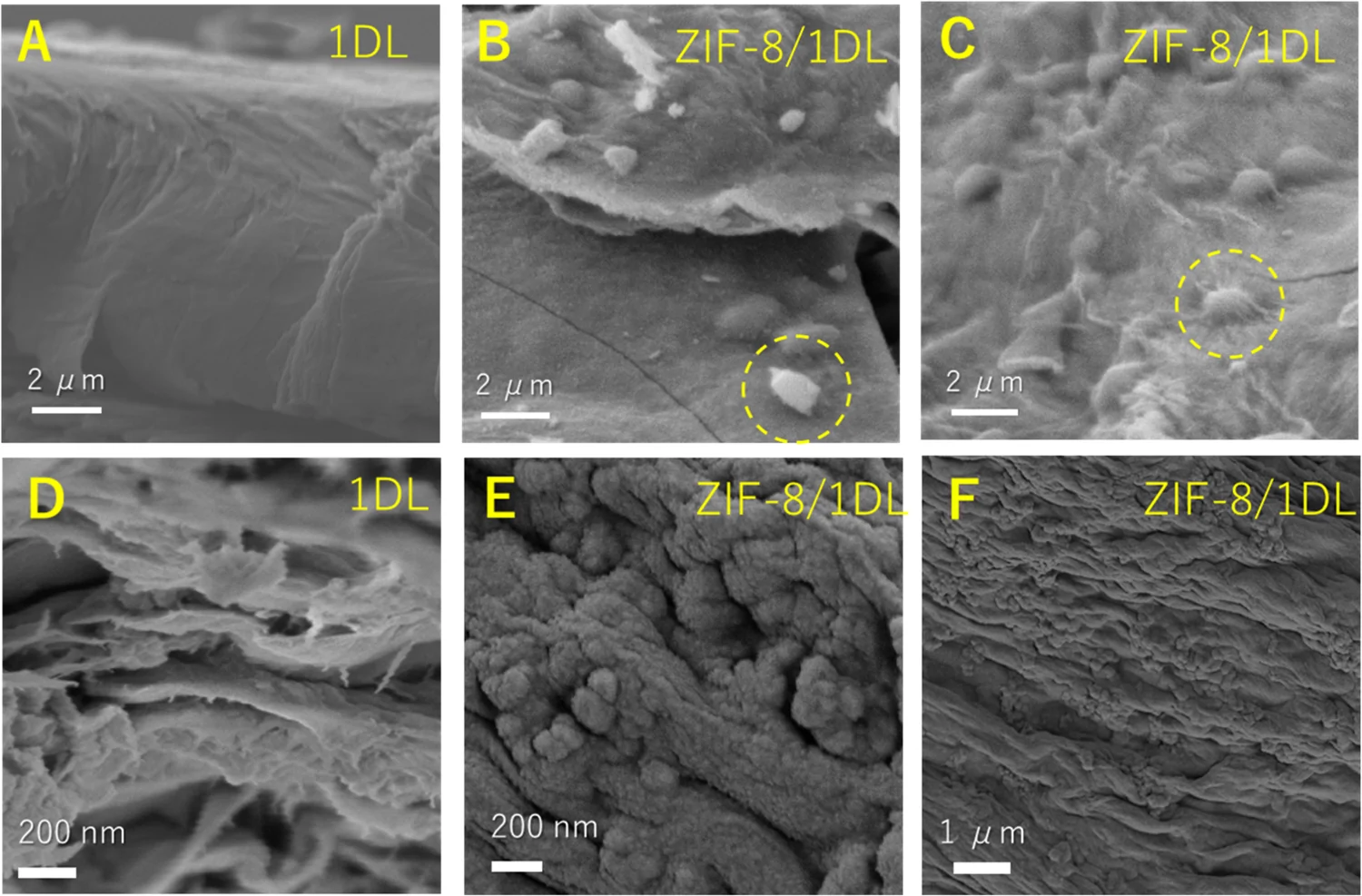
SEM images of gently crushed FFs from water colloids. (A) Surface of 1DL FF. (B) Layers of ZIF-8/1DL FFs. (C) Surface of ZIF-8/1DL FF. (D) Cross section of 1DL FF. (E,F) Cross sections of ZIF-8/1DL FF.

### Adsorption–Desorption Isotherms and Pore-Size Distribution

Figure 6A shows N_2_ adsorption–desorption isotherms at 77 K for materials explored here. The 1DL sample adsorbs a small N_2_ volume (black trace), consistent with limited pore accessibility; magnification (inset) suggests a type IV isotherm with hysteresis, indicative of mesoporosity.^70^ ZIF-8 exhibits a type I isotherm (red trace), as expected for a microporous framework.^56^ The composite (blue trace) combines features of both type IV and type I behavior.

**Figure 6:**
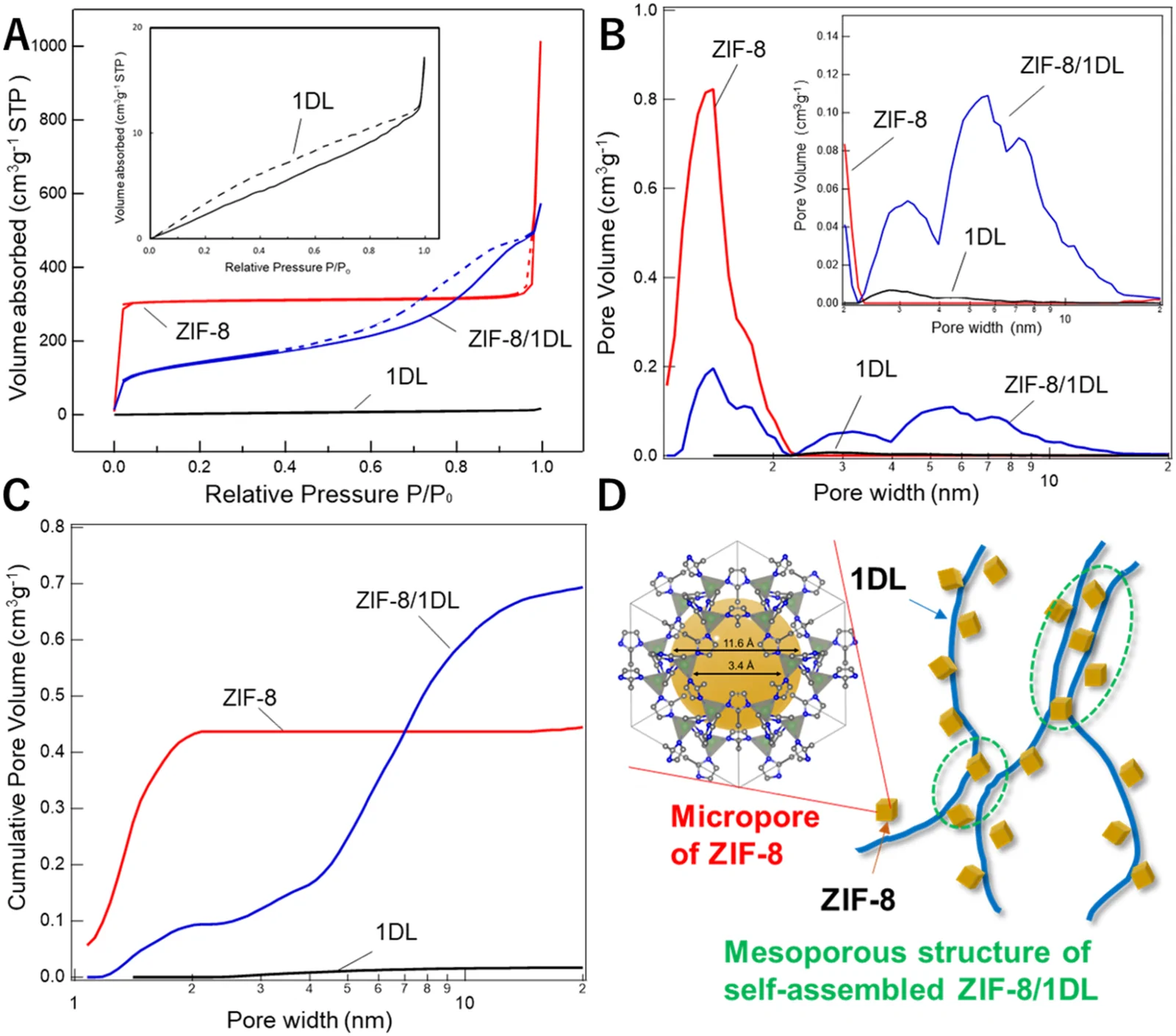
Porosity of ZIF-8/1DL composite and end members at 77 K. (A) N_2_ adsorption–desorption (inset magnifies black 1DL loop). Solid lines: adsorption; dotted lines: desorption. (B) NLDFT pore-volume distributions (inset highlights mesopores). (C) Cumulative pore volume. (D) Schematic of pore formation in the composite.

BET SSAs and pore volumes are summarized in Table 1. The 1DL SSA is 20 m^2^ g^–1^. As noted, low BET values are common in certain nanomaterials (e.g., MXenes) when probe gases do not access interlayer spaces or ultramicropores.^71^ The theoretical area for 2D TiO_2_ sheets ≈0.5 nm thick is ≈500 m^2^ g^–1^, while dye-adsorption studies suggest SSAs ≥1500 m^2^ g^–1^.^21^ The composite SSA increases to ≈500 m^2^ g^–1^ due to the ZIF-8 contribution.

**Table 1:** BET SSA and Pore Volumes for Composites and End Members Tested Herein

Sample	BET SSA (m^2^/g)	Pore volume (cm^3^/g)
1DL	20	0.02
ZIF-8	1297	0.48
ZIF-8/1DL	501	0.72

Pore-size distributions and cumulative pore volumes (Figure 6B,C) further highlight the composite’s uniqueness. ZIF-8 consists predominantly of micropores <2 nm (red curve), whereas the 1DL exhibits limited mesoporosity (2–6 nm; black curve). A recent CO_2_ sorption study indicates that 1DL also possesses ultramicropores.^70^ In the composite (blue curves), both micropores (<2 nm) and mesopores (2–10 nm) are present. A schematic of possible mesopore formation is shown in Figure 6D. The cumulative pore volume of the composite (0.72 cm^3^ g^–1^) exceeds that of ZIF-8 (0.48 cm^3^ g^–1^) and is over an *order of magnitude larger* than that of 1DL (Table 1). It is worth noting that prior studies have created hierarchical MOF–graphene composites with enhanced properties.^72–74^ For example, Jiang et al. prepared graphene/MOF hybrids with improved CO_2_ diffusion,^75^ and Hu et al. reported ultrathin ZIF-8/GO membranes with excellent molecular sieving.^76^

### Electrochemical Evaluation in Lithium–Sulfur Cells

Before presenting the Li–S results we hereby acknowledge that they are quite preliminary and are only included to make the point that the nanocomposite exhibits *improved* Li-ion transport than the end members. To obtain mechanistic information requires much more work that is outside the scope of this paper.

The CV curves for ZIF-8/1DL and 1DL are shown in Figure 7A,B. Standalone ZIF-8 was not tested here because prior reports show poorer capacity than 1DL alone by ≈20–30%.^35,48,77^ According to the literature S@ZIF-8 cathodes initially display high capacities (≈1450 mAh g^–1^ at low current densities) but degrade significantly at 0.5C, dropping to ≈510 mAh g^–1^ by 100 cycles and ≈420 mAh g^–1^ by 200 cycles, attributed to reliance on physical confinement with insufficient chemical binding to suppress shuttling.^35^ In contrast, 1DL-based cathodes maintain ≈750 mAh g^–1^ over 300 cycles with nearly 100% Coulombic efficiency due to Ti^4+^–polysulfide interactions.^48^ Consequently, composites containing 1DL are expected to outperform ZIF-8 alone in long-term cycling and at higher currents.^35,48^

**Figure 7:**
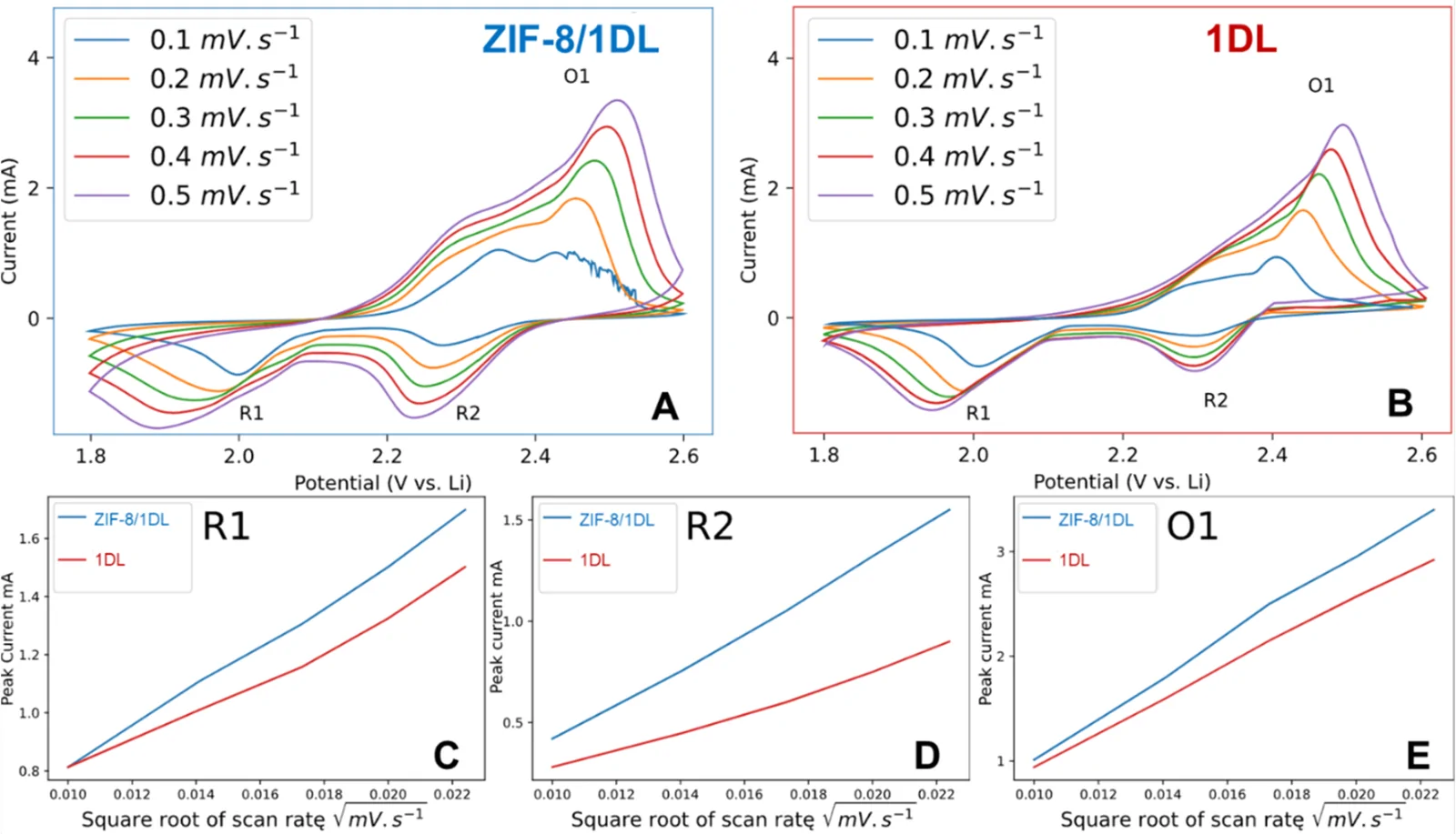
Electrochemical characterization of 1DL and ZIF-8/1DL electrodes in Li–S cells. (A) CV of ZIF-8/1DL; (B) CV of 1DL. in both cases 3 peaks are labelled, R1, R2 and O1. (C–E) Peak-current analyses versus ν^1/2^ for Li^+^ diffusion for 3 peaks labelled on panels

The oxidation peak near 2.5 V (O1; Figure 7A,B,E) corresponds to the oxidation of PSs to elemental S.^78^ The reduction peak near 2.3 V (R2; Figure 7A,B,D) is associated with the conversion of higher-order PSs (Li_2_S_8_ to Li_2_S_5_), and the peak near 2.0 V (R1; Figure 7A,B,C) corresponds to the reduction of Li_2_S_4_/Li_2_S_2_ to Li_2_S.^79^ Peak currents plotted against ν^1/2^ (Figure 7C–E) scale linearly for both electrodes, indicating diffusion-controlled behavior, as described by the Randles–Sevcik equation^79,80^

$$i_{p}=\left(2.69\times 10^{5}\right)n^{1.5}AD_{Li}^{0.5}C_{Li}\nu^{0.5}$$

The higher slopes for the composite indicate enhanced Li^+^ diffusion relative to 1DL alone.

## Conclusions

A hierarchically porous material was developed by combining 1DL with ZIF-8 through a simple one-pot in situ synthesis. This work demonstrates the use of 1DL as a structural framework for MOF-based nanocomposites. The integration of 1DL and ZIF-8 yields nanoscale ZIF-8 particles (≈30 nm) within a 1DL-derived network. Electrostatic interactions between negatively charged 1DL and positively charged ZIF-8 were supported by zeta-potential measurements and corroborated by TEM/EDS mapping, which revealed uniform ZIF-8 dispersion. Nitrogen sorption analyses confirm the presence of hierarchical porosity and increased SSAs relative to 1DL alone. In Li–S batteries, cyclic voltammetry indicates diffusion-controlled behavior with enhanced Li^+^ transport in the composite. These features suggest potential for improved cycling stability and rate capability. Overall, this approach establishes a path to hierarchical porous materials by leveraging the complementary attributes of 1DL and MOFs for energy-storage applications.

## Experimental Methods

### Synthesis of 1DLs

Commercial TiB_2_ powders (−325 mesh, Alfa Aesar, 99.5%) were added to an aqueous tetramethylammonium hydroxide (TMAOH, Alfa Aesar, 25 wt % in water) solution. The Ti:TMAOH molar ratio was maintained at 0.6, consistent with prior work.^5,6^ The mixture was placed in a 60 mL polypropylene bottle and stirred magnetically on a hot plate at 80 °C for 5 days. After reaction, the dark product was washed with ethanol (EtOH, 35 mL, 200 proof, Fisher Scientific) three times and centrifuged at 5000 rpm three times to remove residual TMAOH and organic byproducts. Washing continued until the supernatant reached pH ≈ 7 in EtOH. Deionized (DI) water (40 mL) was then added to disperse the product into a stable 1DL colloid. The dispersion was centrifuged for 0.5 h at 5000 rpm to sediment heavier, larger particles (primarily unreacted precursor). The dark viscous supernatant was either diluted with water or vacuum-filtered to form freestanding films (FFs). A schematic of the process for 1DL FFs and composite powders is shown in Figure 1C.

### Preparation of ZIF-8/1DL Composites

Zinc nitrate hexahydrate (200 mg) and 2-methylimidazole, Hmim, (3.5 g) were dissolved in 40 mL of water to achieve a Hmim:Zn molar ratio of 54:1.^54^ This solution was added dropwise to 40 mL of an aqueous 1DL colloid (≈2 wt %). The mixture was stirred at RT for 24 h.^54^ The product was washed three times with EtOH and twice with DI water to remove excess metal ions and ligands. The resulting gel was subsequently diluted 3-fold with DI water to obtain a colloid that was filtered into a film and dried under vacuum at 80 °C for 3 h. Unless otherwise stated, crushed FFs (prepared by mortar and pestle) were used for characterization.

### Inductively Coupled Plasma Atomic Emission Spectroscopy, ICP-AES

ICP-AES (Thermo Scientific iCAP 6300 Radial) was used to determine the Zn/Ti ratio. Crushed FF powders were digested by μ-WAVE and analyzed. Values are reported as quantitative estimates based on concentrations measured using a two-point calibration curve with specified standards.

### X-ray Diffraction, XRD

XRD patterns were acquired using a Rigaku MiniFlex diffractometer (Cu Kα radiation, 40 kV, 15 mA). Crushed FFs were scanned over 2θ = 2–65° with a 0.02° step and 1 s dwell time. Unless otherwise noted, powders were dried at 50 °C overnight before scanning. Mean crystallite size (τ) was estimated using the Scherrer equation, τ = (Kλ)/(β cos θ), with *K* = 0.95 for near-spherical particles; β is FWHM line broadening and λ is the X-ray wavelength. Instrumental peak-shape parameters were obtained using a LaB_6_ standard (NIST 660B). Profile fitting employed constrained least-squares refinement of intensity, FWHM, and 2θ values. The residual *R*_p_ quantifies goodness of fit.

### Scanning Electron Microscopy

A scanning electron microscope, SEM, (Zeiss Supra 50 VP with in-lens detector, 30 mm aperture, 3–5 kV) was used to image the powders. Samples were prepared from gently crushed FFs.

### Transmission Electron Microscopy

A high-resolution transmission electron microscope, HRTEM, (JEOL F200) was used to obtain micrographs and selected-area electron diffraction (SAED) patterns. Samples were prepared by diluting crushed FFs 1000× in water and drop-casting onto Cu grids. ImageJ was used for postprocessing of TEM and SAED data.

### X-ray Photoelectron Spectroscopy, XPS

XPS spectra were collected on a VersaProbe 5000 (Physical Electronics) using monochromatic Al Kα radiation (pass energy 23.5 eV, step size 0.05 eV, spot size 200 μm). Crushed FF powders were mounted with carbon tape. CasaXPS v2.3.21PR1.0 was used for peak fitting and quantification with a Shirley background. Spectra were charge-corrected by setting the C–C peak to 285.0 eV.

### Zeta Potential and Particle Size Measurements

Electrophoretic mobility was measured on a Nano-ZS (Malvern Panalytical) and converted to zeta potential via the Smoluchowski model. All measurements were conducted at ambient conditions with a 120 s equilibration. Hydrodynamic diameters were measured using a NanoBrook Omni (Brookhaven Instruments). Colloids at pH ≈ 7 were placed in quartz cuvettes and equilibrated for 30 s before measurement.

### Gas Sorption

N_2_ adsorption–desorption measurements at 77 K were performed on a Quantachrome Autosorb iQ. Samples (crushed FFs) were degassed under vacuum at 60 °C for 1 h, 100 °C for 1 h, and 150 °C for 12 h. Specific surface areas were calculated using the BET method. Pore-size distributions were obtained by nonlocal density functional theory (NLDFT).

### Post Treatment of 1DL and ZIF-8/1DL for Li–S Cells

Colloidal suspensions of 1DL or ZIF-8/1DL were mixed with 5 M aqueous LiCl (anhydrous, 99%, 20 mesh, Alfa Aesar) at twice the colloid volume and stirred overnight. The suspension was washed with DI water and centrifuged to neutral pH, then vacuum-filtered and freeze-dried (liquid N_2_) overnight. The resulting powder was mixed with S at a 1:1 mass ratio and heated in a tube furnace from room temperature to 155 °C at 2 °C min^–1^, held for 12 h, and cooled to RT.

### Preparation of Electrodes for Li–S Cells

Composite slurries were prepared as described previously.^48^ The slurry comprised 70 wt % (1DL or ZIF-8/1DL with sulfur), 20 wt % Super P carbon black, and 10 wt % PVDF binder (battery grade). Components were mixed in a planetary mixer (Thinky) at 2000 rpm for 8 min with 500 μL *N*-methyl-2-pyrrolidone and zirconia mixing balls. The slurry was cast on aluminum foil using an automatic casting table (MTI). Coated electrodes were dried at 50 °C for 6 h under vacuum, cooled to RT, and subsequently freeze-dried for 24 h.

### Li–S Coin Cells

Circular cathodes (11 mm) were punched from dried electrodes and transferred to an Ar-filled glovebox (O_2_ and H_2_O < 1 ppm, Mbraun Labstar). The S loading was ≈1 mg per electrode. Coin cells (CR2032; MTI and Xiamen TMAX) were assembled with Celgard 2325 separators. The electrolyte was 1 M LiTFSI with 1 wt % LiNO_3_ in a 1:1 (v:v) mixture of 1,2-dimethoxyethane and 1,3-dioxolane (Gotion). Lithium metal anodes (13 mm disks, Alfa Aesar) were used. The electrolyte-to-sulfur ratio was 20 μL mg^–1^. Cells rested for 10 h (average OCV ≈ 2.3 V). Cyclic voltammetry was performed on a VMP3 potentiostat (Biologic) between 1.8 and 2.6 V vs Li/Li^+^ at scan rates of 0.1–0.5 mV s^–1^.

## Supplementary Material



## Notes

Weight and molar ratio of ZIF-8 and 1DL composite; XRD patterns for ZIF-8/1DL composite and average particle size determined from the Scherrer equation; zeta potential distributions; SAED patterns of ZIF-8/1DL composite; TEM micrographs of 1DL alone; schematic of 1DL-assisted *in situ* synthesis of ZIF-8 and self-assembly into 2D sheets (PDF)
