# Supplementary material for: Quantum Confined One-Dimensional Lepidocrocite TiO2–Assisted In Situ Synthesis of ZIF‑8 MOF: Hierarchical Porous Nanocomposites Exhibit Improved Li-ion Transport
Source: ACS Mater Au. 2026 Oct 16;6(6):1399–409. doi: 10.1021/acsmaterialsau.6c00110 (PMC13760626; doi:10.1021/acsmaterialsau.6c00110)
Supplement: Supplementary file 1 [file mg6c00110_si_001.pdf]

Supporting Information

Quantum Confined One-dimensional Lepidocrocite TiO_2-Assisted in-situ Synthesis of ZIF-8 MOF: Hierarchical Porous Nanocomposites Exhibit Improved Li-ion Transport

Takayuki Kono,[1]‡ Avishek Karmakar,[2]‡ Keita Tsubakimoto,[1] Kaustubh Sudhakar,[2] Neal A. Cardoza,[3] Vibha Kalra,[3,4] Tarek Ali ElMelegy,[2,5], Kota Ota,[1] Mary Q. Hassig,[2] Takashi Kanazawa,[1] Hiroyuki Morita,[1] Shun Sakaida,[1] Takeshi Torita,[1] and Michel W. Barsoum[2]**

[1]Murata Manufacturing Co., Ltd., Nagaokakyo-shi, Kyoto, Japan

[2]Department of Materials Science and Engineering Drexel University, Philadelphia, Pennsylvania 19104, United States

[3]Department of Chemical and Biological Engineering, Drexel University, 3141 Chestnut Street, Philadelphia, Pennsylvania 19104, United States

[4] Smith School of Chemical and Biomolecular Engineering, Cornell University, Ithaca, New York 14853, United States.

[5] Department of Mechanical Engineering, The British University in Egypt, Cairo, Egypt.

*E-mail: t_torita@murata.com; barsoumw@drexel.edu

Table S1. Weight and molar ratio of ZIF-8 and 1DL in composite estimated from ICP-AES.

Material	Weight ratio (%)		Weight ratio (%)		Molar ratio (%)	
	Zn	Ti	$C_8H_{12}N_4Zn$	TiO_2	$C_8H_{12}N_4Zn$	TiO_2
ZIF-8/1DL (22:78 wt%)	4.2	31.0	21.9	78.1	8.9	91.1

Average crystallite size of the ZIF-8 nanoparticles in composite

The average crystallite size of the ZIF-8 nanoparticles within the composite matrix was determined using the Scherrer equation:

$$D = \frac{K\lambda}{\beta \cdot cos\theta} \quad \text{(S1)}$$

where D is the mean crystallite size, K is the dimensionless shape factor (0.95, assuming spherical crystallites with cubic symmetry), λ is the X-ray wavelength of Cu Kα radiation (1.5418 Å), β is the full-width at half-maximum (FWHM) of the diffraction peaks in radians after correcting for instrumental broadening, and θ is the Bragg angle in radians.

To ensure accurate crystallite size determination, the instrumental broadening was evaluated using a NIST-certified lanthanum hexaboride (LaB_6, SRM 660c) standard. The instrumental broadening function was determined by analyzing LaB_6 peaks across the measured 2θ range, and the extracted FWHM values were corrected accordingly. Peak fitting was performed using an Intermediate Lorentzian (IL) function.

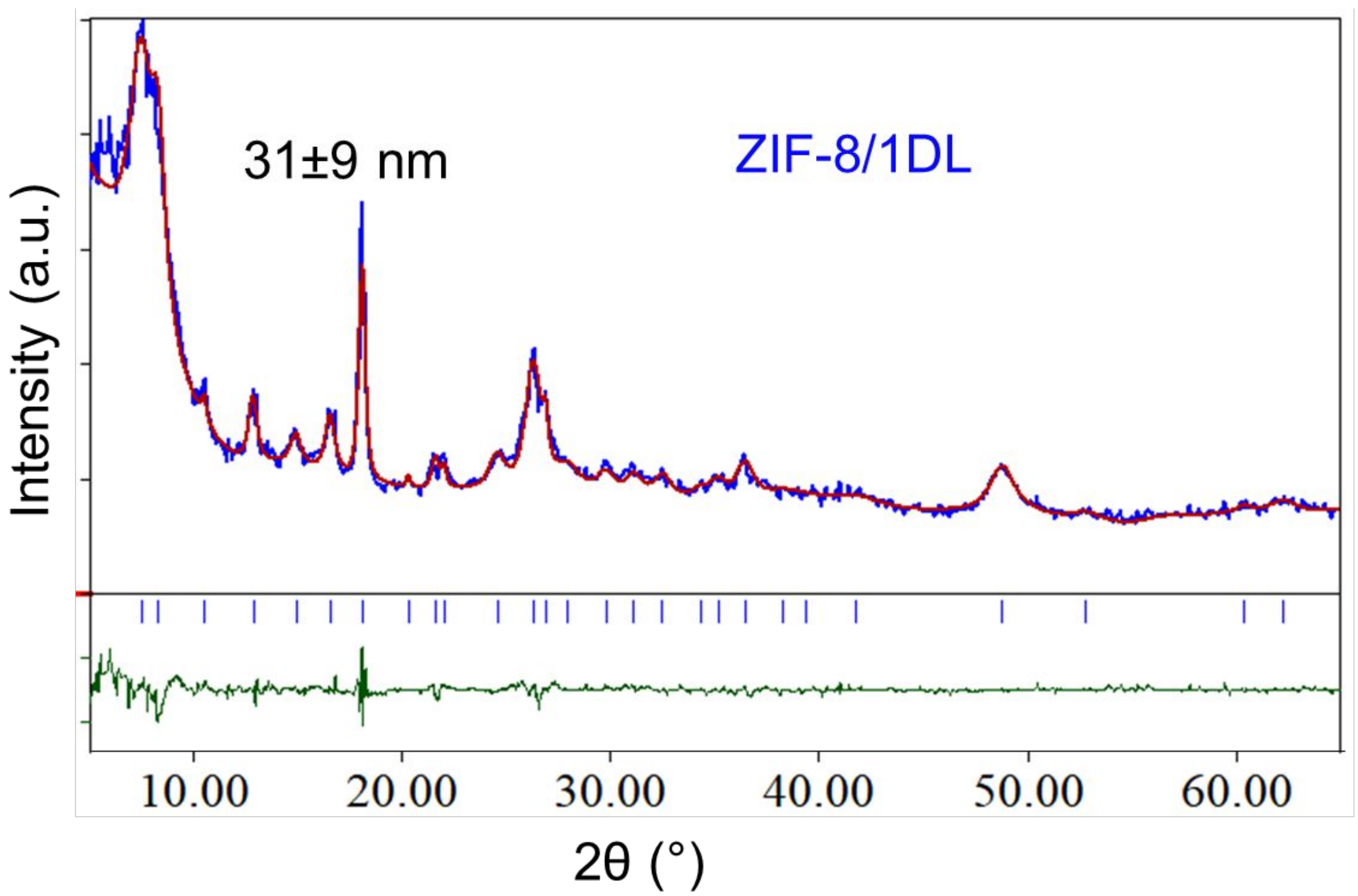

Figure S1. XRD patterns for ZIF-8/1DL composite. Fit to the data in blue, is denoted in red. Differences between experimental and fits are shown in green. Average particle size, in nm, determined from the Scherrer formula is also shown on panels, it calculated from the peaks at 10.4˚, 12.7˚, 14.9˚, 16.5˚ and 18.2˚ whereas the peak at 7.4˚ is skipped since it was overlapped with the (020) of 1DL.

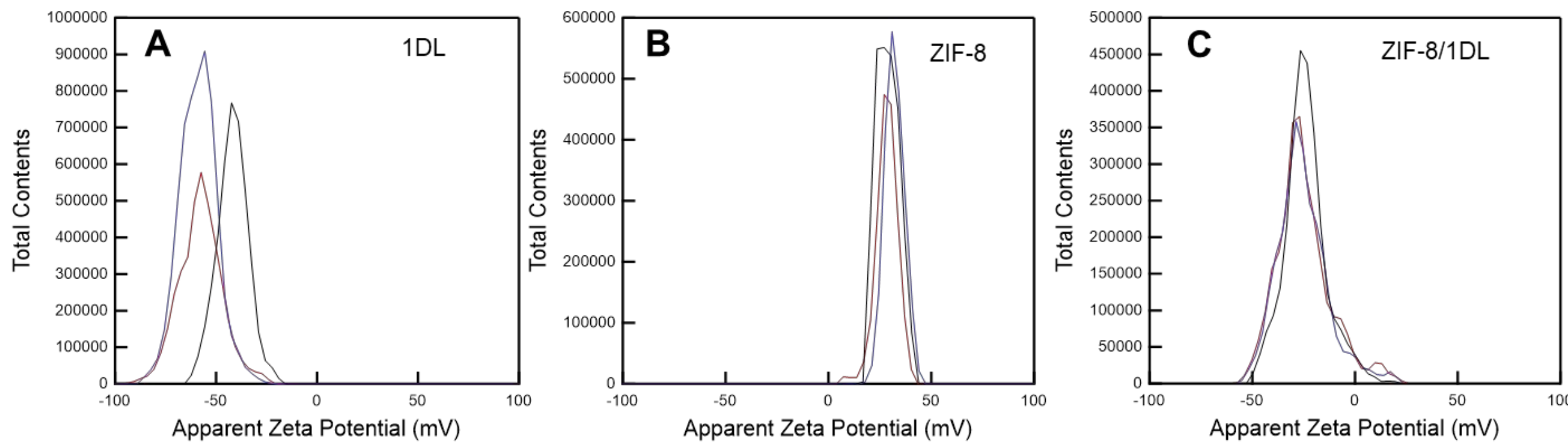

Figure S2. Zeta potential distributions, total counts versus zeta potential (mV) of, (A) 1DL, (B) ZIF-8, (C) ZIF-8/1DL. All values were measured 3 times.

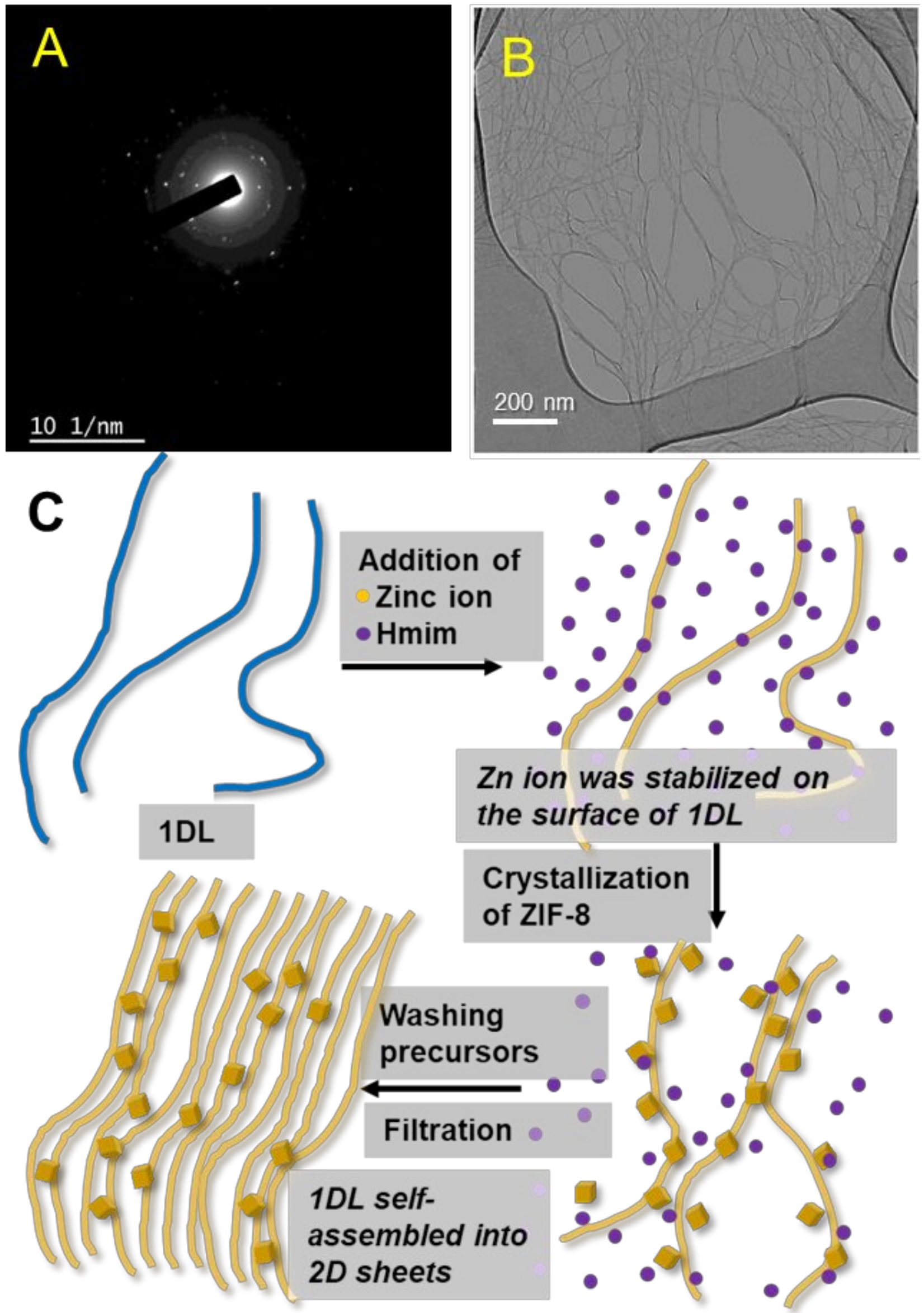

Figure S3: (A) SAD corresponding to TEM micrographs shown in Fig. 4C. Samples were prepared from filtered films, crushed into powders, diluted 1000 X in water. (B) Typical TEM images of 1DL. (C) Schematic of 1DL-assisted in-situ synthesis of ZIF-8 and self-assembling into 2D sheets.
